# Augmentation of partially regenerated nerves by end-to-side side-to-side grafting neurotization: experience based on eight late obstetric brachial plexus cases

**DOI:** 10.1186/1749-7221-1-6

**Published:** 2006-12-05

**Authors:** Sherif M Amr, Ashraf N Moharram, Kamal MS Abdel-Meguid

**Affiliations:** 1 From the Department of Orthopaedics and Traumatology, Cairo University, Cairo, Egypt; 2 From the Department of Orthopaedics and Traumatology, Fayoum University, Fayoum, Egypt

## Abstract

**Objective:**

The effect of end-to-side neurotization of partially regenerated recipient nerves on improving motor power in late obstetric brachial plexus lesions, so-called nerve augmentation, was investigated.

**Methods:**

Eight cases aged 3 – 7 years were operated upon and followed up for 4 years (C5,6 rupture C7,8T1 avulsion: 5; C5,6,7,8 rupture T1 avulsion:1; C5,6,8T1 rupture C7 avulsion:1; C5,6,7 ruptureC8 T1 compression: one 3 year presentation after former neurotization at 3 months). Grade 1–3 muscles were neurotized. Grade0 muscles were neurotized, if the electromyogram showed scattered motor unit action potentials on voluntary contraction without interference pattern. Donor nerves included: the phrenic, accessory, descending and ascending loops of the ansa cervicalis, 3^rd ^and 4^th ^intercostals and contralateral C7.

**Results:**

Superior proximal to distal regeneration was observed firstly. Differential regeneration of muscles supplied by the same nerve was observed secondly (superior supraspinatus to infraspinatus regeneration). Differential regeneration of antagonistic muscles was observed thirdly (superior biceps to triceps and pronator teres to supinator recovery). Differential regeneration of fibres within the same muscle was observed fourthly (superior anterior and middle to posterior deltoid regeneration). Differential regeneration of muscles having different preoperative motor powers was noted fifthly; improvement to Grade 3 or more occurred more in Grade2 than in Grade0 or Grade1 muscles. Improvements of cocontractions and of shoulder, forearm and wrist deformities were noted sixthly. The shoulder, elbow and hand scores improved in 4 cases.

**Limitations:**

The sample size is small. Controls are necessary to rule out any natural improvement of the lesion. There is intra- and interobserver variability in testing muscle power and cocontractions.

**Conclusion:**

Nerve augmentation improves cocontractions and muscle power in the biceps, pectoral muscles, supraspinatus, anterior and lateral deltoids, triceps and in Grade2 or more forearm muscles. As it is less expected to improve infraspinatus power, it should be associated with a humeral derotation osteotomy and tendon transfer. Function to non improving Grade 0 or 1 forearm muscles should be restored by muscle transplantation.

**Level of evidence:**

Level IV, prospective case series.

## Background

Late obstetric brachial plexus palsy serves as a good example for studying the outcome of partially regenerated nerves. Three main types of lesion [1] have been recognized. In a C5-6 lesion, the arm is adducted and internally rotated at the shoulder and the elbow extended. The forearm is pronated and the wrist (and sometimes fingers) flexed. In a C5-7 lesion, in addition to the above, the elbow may be slightly flexed. In a C5-T1 lesion, the arm is totally flail with a claw hand. In a prospective study of 80 infants with brachial plexus injury followed up for more than 4 years [2], complete recovery occurred in 66% of cases; mild weakness persisted in 11%, moderate arm weakness in 9% and 14% had severe permanent weakness. This unfavourable prognosis was supported by others [3]. Several schemes were suggested to establish the natural history of the injury selecting those cases not expected to recover for early surgery [1]. Although early surgery was advocated [4], in C5-7 lesions the shoulder and elbow did not do as well as in upper-type lesions, the results at the level of the hand were encouraging, however, showing 75% with useful function after 8 years [5,6]. In a further study [7], good results were obtained in 33% of C5 repairs, in 55% of C6, in 24% of C7 and in 57% of operations on C8 and T1. Posterior dislocation of the shoulder was observed in 30 cases. All were successfully relocated after the age of one year. A residual shoulder internal rotation deformity requiring secondary surgery was also noted by others [8]. Thus, with or without early surgery, a residual disability remains. This disability increases with age [9], necessitating surgical correction.

For correcting residual shoulder internal rotation adduction, humeral derotation osteotomies [10] or tendon transfers [11] gave good results. Nevertheless, this can only occur if there is some range of shoulder abduction. Besides, the early satisfactory results of anterior release and latissimus dorsi to rotator cuff transfer are not maintained. In one study [12], there was loss of active external rotation, because of gradual degeneration of the transferred muscles, contracture of the surrounding soft tissues and degenerative changes in the glenohumeral joint. In another study [13], children with sequelae of C5-C6 palsy gained in abduction and external rotation more than children with C5-C6-C7 or complete palsy. Patients with mild preoperative shoulder dysfunction achieved the best results. The clinical results were related to the type of paralysis and to preoperative shoulder function, but not to age at surgery. Progressive deterioration of abduction began at 6 years despite preserved active external rotation. In a prospective study of secondary surgery on 183 subluxations or dislocations of the shoulder consequent upon obstetric brachial plexus palsy [14], 20 failures were reported. The functional outcome was related to the severity of the neurological lesion, the duration of the dislocation and onset of deformity.

Apart from the shoulder, corrective surgery would not benefit a forearm or hand which had regained little function and might have remained flail.

The conclusion is, in many cases muscle power has to be improved before embarking on secondary reconstructive procedures.

The technique of (recipient)end-to-(donor)side neurorrhaphy [15] allowed neurotization of injured nerves without affecting donor nerves. Reverse end-to-side neurotization [16] allowed neurotization of partially injured recipient nerves without downgrading already regained recipient muscle power, a technique which we called nerve augmentation. This was tried out experimentally [17]. It was also carried out in early complete obstetric brachial plexus palsy [18]. In a previous work [19], we introduced several end-to-side side-to-side neurorrhaphy techniques, which made it easier to tackle this problem.

In this study and using the latter techniques, we aim to investigate the effect of nerve augmentation on improving motor power in late obstetric brachial plexus lesions.

## Materials and methods

### Patients

8 patients suffering from obstetric brachial plexus palsy were operated upon from 1996 up to 2001 and followed up for 4 years.

Their ages at the time of surgery ranged from 3 up to 7 years with a median of 4 years; 1 was male, the rest female.

5 patients were late presentations of a C5,6 rupture C7,8T1 avulsion, 1 was a late presentation of a C5,6,7,8 rupture T1 avulsion, 1 was a late presentation of a C5,6,8T1 rupture C7 avulsion; the eighth patient presented to us 3 years after having been operated upon at the age of 3 months, when sural and radial nerve grafting had been carried out for a C5,6,7 rupture, C8 T1 neurolyzed.

The demographic data, clinical and operative findings and operative procedures are presented in Table 1.

**Table 1:** The demographic data of the patients, lesion types, operative procedures, preoperative cocontractions and deformities and the pre- and postoperative evaluation scores.

Pt	Age sex	Type of Lesion	Procedure	Cocontractions	Deformities				Nerve grafts	Shoulder function score		Elbow function score		Hand function score	
			Donor to recipient		shoulder	elbow	forearm	Wrist		Preop. Postop.		Preop. Postop.		Preop. Postop.	
1	4F	C5,6 rupture C7,8T1 avulsion	Phrenic to suprascapular; contralateral C7 to all cords	**Cocontractions **of biceps, clav. pect. major and deltoid on shoulder abduction and elbow flexion	**Internal rotation **adduction (+ve scapular elevation sign)	Flexion deformity 20 degrees	**Supination def**.	Flexible extension deformity	Sural and radial ns.	2	4	3	4	1	2
2	4F	C5,6,7,8 rupture T1 avulsion	Phrenic to suprascapular; contralateral C7 to all cords	Cocontractions of biceps and deltoid on shoulder abduction and elbow flexion	Internal rotation adduction (+ve scapular elevation sign)	Flexion deformity 30 degrees	Pronation def.	**Wrist drop**	sural	2	2	2	3	1	3
3	7M	C5,6 rupture C7,8T1 avulsion	Ansa cervicalis to musculocutaneous and median, phrenic to axillary, spinal accessory to suprascapular	-	-	Flexion deformity 10 degrees	**Supination def.**	Flail wrist	sural	2	4	4	5	1	1
4	4 F	C5,6 rupture C7,8T1 avulsion	Spinal accessory to axillary, Phrenic to ulnar, Ansa cervicalis to radial	-	Internal rotation adduction (+ve scapular elevation sign)	-	-	-	sural	5	5	4	4	4	4
5	6 F	C5,6,8T1 rupture C7 avulsion	Phrenic to suprascapular; contralateral C7 to all cords	-	-	Flexion deformity 10 degrees	**Supination def.**	Flexible flexion deformity	sural	5	5	5	5	5	5
6	3 F	C5,6 rupture C7,8T1 avulsion	Phrenic to suprascapular; contralateral C7 to all cords	Cocontractions of biceps and deltoid on shoulder abduction	**Internal rotation **adduction (+ve scapular elevation sign)	Flexion deformity 20 degrees	**Supination def**	Flail wrist Flexible extension deformity	sural	2	4	4	4	1	2
7	4 F	C5,6 rupture C7,8T1 avulsion	Phrenic to suprascapular; contralateral C7 to all cords	**Cocontractions **of biceps, deltoid and wrist extensors on shoulder abduction and elbow flexion	**Internal **rotation adduction (+ve scapular elevation sign)	Flexion deformity 20 degrees	**Supination def**	Flexible extension deformity	sural	4	6	3	4	1	2
8	3 F	sural and radial nerve grafting for C5,6,7 rupture, C8 T1 neurolysis; at the age of 3 months	3^rd ^and 4^th ^intercostals to musculocutaneous n. (intertwining neurotization); partial ulnar to radial n. interwining neurotization (mod. Oberlin transfer); Ulnar to median (side-to-side neurotization); external rotation osteotomy and Hoffer transfer (lat. dorsi and teres major tendons to infraspinatus)	**Cocontractions **of biceps and deltoid on elbow flexion	**Internal **rotation adduction (+ve scapular elevation sign)	Flexion deformity 10 degrees	-	Flexible flexion deformity 10 degrees	-	4	6	5	5	5	5

### Patient evaluation

All patients were evaluated pre- and postoperatively (every 6 months) for deformities, muscle function, cocontractions and upper limb growth. To limit intraobserver and interobserver variability, testing for deformities, muscle function and cocontractions was recorded by digital photography on both normal and healthy sides. The normal side was recorded to ensure the patient had complied with the examiner's instructions. Electromyographic studies and cervical myelography were performed preoperatively. Root avulsions were evaluated by CT cervical myelography [20] and confirmed intraoperatively [21]. Shoulder, elbow and hand functions were scored pre- and postoperatively using the modified Gilbert shoulder evaluation scale, the Gilbert elbow evaluation scale and the hand evaluation scale according to Raimondi respectively [22].

### Deformities

At the shoulder, 6 patients had an internal rotation adduction deformity with a positive Putti's scapular elevation sign. At the elbow, 3 had a 20 degree flexion deformity, 2 a 10 degree flexion deformity, 1 a 30 degree flexion deformity. At the forearm, 5 had a supination deformity and 1 a pronation deformity. At the wrist, 2 had a flail wrist, 2 a flexible flexion deformity with preservation of some wrist extension, 1 a complete wrist drop and 2 a flexible extension deformity. Deformities in individual patients are shown in Table 1.

### Muscle function

Muscle function was assessed using the system described in the report of the Nerve Committee of the British Medical Council in 1954 and previously used by other authors [23]. Muscle testing was complicated by the presence of cocontractions and deformities. The highest muscle power value was taken regardless of cocontractions.

In testing the shoulder muscles, we faced the following problems. First, the anterior, middle and posterior deltoid had to be tested separately [24]. The second problem was testing for the subscapularis, which is usually tested by the lift-off test and the lift-off lag sign [25-27]. Using both of the above tests was difficult both because of cocontractions between the anterior and lateral parts of the deltoid and the biceps muscle on elbow flexion and because of the absence of shoulder extension. The belly press (Napoleon) test was more applicable in our cases. Identifying a sensitive test for supraspinatus function was the third problem. This was done using Jobe's empty can test. Identifying a sensitive test for infraspinatus function was the fourth problem. Infraspinatus integrity is usually tested by the external rotation lag (dropping) sign, by Hornblower's sign and by the drop arm sign. These tests were modified to test for muscle power. Although all of the above tests were reliable, the most sensitive test was the drop arm test [25]. Some reports questioned its sensitivity, however [27]. In the current study, when the patient could actively abduct his shoulder, the drop arm sign was used, as it was the most sensitive; otherwise, the other two tests were used.

In testing finger flexors and extensors, both elbows and wrists were immobilized on a board.

### Evaluation for cocontractions

Cocontractions were evaluated by asking the patient to flex the shoulder without actively abducting, internally or externally rotating it and without actively moving the elbow, forearm, wrist or fingers. He was observed if he could flex the shoulder independently of other movements. The same procedure was repeated for shoulder abduction, elbow flexion and extension, forearm pronation and supination, wrist and finger flexion and extension. Cocontractions of the biceps and deltoid both on shoulder abduction and on elbow flexion were present in 3 cases; in Case2 without any other cocontractions, with additional cocontractions of the clavicular head of the pectoralis major in Case1, and with additional cocontractions of the wrist extensors in Case7 (Table 1). Cocontractions of the biceps and deltoid on shoulder abduction only was noted in Case6. Cocontractions of the biceps and deltoid on elbow flexion only was also noted in Case8.

### Evaluation scales

The Gilbert shoulder scale comprised the following grades: Grade 0: completely paralysed shoulder or fixed deformity; Grade 1: abduction = 45 degrees, no active external rotation; Grade 2: abduction < 90 degrees, bi-active external rotation; Grade 3: abduction = 90 degrees, active external rotation < 30 degrees; Grade 4: abduction < 120 degrees, active external rotation 10–30 degrees; Grade 5: abduction > 120 degrees, active external rotation 30–60 degrees; Grade 6: abduction > 150 degrees, active external rotation > 60 degrees).

The Gilbert elbow scale included the following items: flexion (1: no or minimal muscle contraction, 2: incomplete flexion, 3: complete flexion); extension (0: no extension; 1: weak extension; 2: good extension); flexion deformity (extension deficit) (0: 0–30 degrees, -1:30–50 degrees, -2:> 50 degrees). Evaluation was as follows: 4–5 points: good regeneration; 2–3 points: moderate regeneration; 0–1 points: bad regeneration

The Raimondi hand evaluation scale comprised the following grades: Grade 0: complete paralysis or minimal useless finger flexion; Grade 1: useless thumb function, no or minimal sensation, limitation of active long finger flexors; no active wrist or finger extension, key-grip of the thumb; Grade 2: active wrist extension; passive long finger flexors (tenodesis effect); Grade 3: passive key-grip of the thumb (through active thumb pronation), complete wrist and finger flexion, mobile thumb with partial abduction, opposition, intrinsic balance, no active supination; Grade 4: complete wrist and finger flexion, active wrist extension, no or minimal finger extension, good thumb opposition with active intrinsic muscles (ulnar nerve), partial pronation and supination; Grade 5: as in Grade 4 in addition to active long finger extensors, almost complete thumb pronation and supination.

### Selection for surgery

All nerves to muscles with motor power less than 4 were selected for neurotization. The axillary nerve was neurotized if the anterior deltoid had a motor power 4, but the lateral and posterior deltoids had motor powers less than 4. The suprascapular nerve was neurotized if the supraspinatus had a motor power 4, but the infraspinatus a motor power less than 4. Nerves to muscles with motor power 0 were also neurotized, if the electromyogram showed scattered motor unit action potentials on voluntary contraction without interference pattern. This was arbitrarily taken as a sign that the muscle bulk had not been completely replaced by fibrosis and therefore function might be restored to it.

### Operative procedure

In the first 7 cases, the brachial plexus was approached through a transverse supraclavicular incision with a deltopectoral extension, yet without clavicular osteotomy [27]. After cutting the clavicular head of the sternomastoid and the insertion of scalenus anterior muscle medially, and the clavicular and part of acromial insertion of the trapezius muscle laterally [28,29], exploration of the brachial plexus proceeded as described elsewhere [21,30-32].

In Cases 1,2, 5, 6, 7, the intranervous intertwining technique [19] was used to neurotize the phrenic nerve (donor) to the suprascapular nerve without nerve grafts. The long length contact technique [19] was used to neurotize the ventral part of contralateral C7 to the lateral and medial cords and the dorsal part of contralateral C7 to the posterior cord [21]. Nerve grafts were laid in a postoesophageal premuscular plane [33] to shorten the distance between contralateral C7 and the recipient plexus. Both sural nerves and the superficial radial nerve served as nerve grafts.

In Case 3, the inferior part of the spinal accessory nerve was located on the anterior surface of the trapezius muscle after cutting its insertion to the clavicle and acromion process and reflecting it posteriorly [28,29]. The intranervous intertwining technique [19] was used to neurotize this donor nerve to the suprascapular nerve without nerve grafts. The phrenic nerve (donor) was neurotized to the axillary nerve via closed loop grafting [25]. The descending and ascending loops of the ansa cervicalis (donor) were exposed on the anterior surface of the internal jugular vein, followed to the superior and inferior bellies of the omohyoid muscle and neurotized to the musculocutaneous and median nerves via side grafting neurrorhaphy.

Similarly, in Case 4, the intranervous intertwining technique [19] was used to neurotize the spinal accessory nerve (donor) to the axillary reinforced by side grafts, and the phrenic nerve to the ulnar without grafts. The ansa cervicalis (donor) was neurotized to radial nerve via side grafting neurrorhaphy.

Case 8 had been successfully explored before via the supraclavicular route. To compensate for the residual internal rotation adduction contracture of the shoulder and its weak external rotation, an external rotation humeral osteotomy and a Hoffer transfer (latissimus dorsi and teres major tendons to the infraspinatus tendon) were performed. An anterior axillary axillary route was chosen both for the above procedure and for subsequent neurotization. The intranervous intertwining technique [19] was used to neurotize the 3^rd ^and 4^th ^intercostal nerves (donors) to the musculocutaneous nerve without nerve grafts. In a modified Oberlin transfer [34] the dorsolateral part of the ulnar nerve was intertwined through the radial nerve. Next side-to-side neurotization of the ulnar to the median nerve was carried out.

## Results

Improvements in motor power are shown in Table 2 and could be summarized as follows.

**Table 2:** The pre- and postoperative motor power grades of the individual muscles in each patient, their median, minimum and maximum values and their range

Pt	Bi = ceps		Deltoid						Rotator cuff ms.						Pectoralis major				Lat. dorsi		Triceps		Fore = arm pron.		Fore = arm sup.		Wrist extensors (extrs.)				Wrist flexors				Finger extrs.		Finger		flexors		Thumb								Intrinsic muscles			
			ant		lat		post		Supra = Spin = atus		Infra = spin = atus		Sub = scap = ularis		Clav. head		Pect. head						Pron. teres		Supi = nator		Ulnar (ECU)		Radial (ECRL & br.)		Ulnar (FCU)		Radial (FCR)				FDS to Ds2-5		FDP to Ds2-5		FPL		EPL		EPbr.		Abd. Poll.		Suppl. by ulnar n.		Suppl. by median n.	
	C5,6		C5,6		C5,6		C5,6		C(4),5,6		C(4),5,6		C5,6,(7)		C5,6		C7,8T1						C6,7		C5,6		C7,8		C6,7–C7,8		C7,8		C6,7		C7,8		C7,8T1		C8T1		C8T1		C7,8		C7,8		C7,8		C8T1		C7,8	
	pre/post		pre/post		pre/post		pre/post		pre/post		pre/post		pre/post		pre/post		pre/post		pre/post		pre/post		pre/post		pre/post		pre/post		pre/post		pre/post		pre/post		pre/post		pre/post		pre/post		pre/post		pre/post		pre/post		pre/post		pre/post		pre/post	
1	3	5	3	5	2	4	0	2	3	4	0	2	2	4	3	4	3	4	4	4	2	3	0	2	0	0	1	3	0	0	0	2	0	0	1	3	1	2	1	2	0	0	1	2	0	0	0	0	0	0	0	0
2	3	4	3	3	2	3	0	0	3	4	0	0	2	3	3	4	3	4	4	4	2	3	0	2	0	0	0	1	0	0	2	4	0	0	0	0	1	3	1	3	0	0	0	1	0	0	0	0	2	3	1	3
3	3	5	3	5	2	4	0	2	3	4	0	2	2	4	3	4	3	4	4	4	4	4	0	2	0	0	0	0	0	0	4	4	3	3	0	0	3	3	1	1	2	2	3	3	0	0	0	0	0	0	0	0
4	3	4	2	4	2	4	2	3	3	4	2	2	2	3	3	4	3	4	4	4	2	2	0	2	0	0	2	3	0	0	0	0	2	3	**Ds** **2,3:** **3** **Ds** **4,5:** **0**	**Ds** **2,3:** **4** **Ds** **4,5:** **0**	**Ds** **2,3:** **3** **Ds** **4,5:** **0**	**Ds** **2,3:** **4** **Ds** **4,5:** **0**	**Ds** **2,3:** **3** **Ds** **4,5:** **0**	**Ds** **2,3:** **4** **Ds** **4,5:** **0**	2	3	0	0	0	0	0	0	0	0	0	0
5	3	4	3	5	3	4	2	4	4	4	3	4	4	4	4	4	4	4	4	4	3	4	0	2	2	2	2	4	2	4	2	4	2	4	2	3	3	3	3	3	3	3	3	2	3	3	2	2	2	2	2	2
6	5	5	3	5	2	4	0	2	3	4	2	2	2	4	3	4	3	4	4	4	2	3	0	2	0	0	1	3	0	0	0	0	0	0	1	3	1	2	1	2	0	0	1	2	0	0	0	0	0	0	0	0
7	3	5	3	5	2	4	0	2	3	4	0	3	2	4	3	4	3	4	4	4	2	3	0	2	0	0	1	3	0	0	0	0	0	0	0	1	0	2	0	2	0	0	0	0	0	0	0	0	0	0	0	0
8	4	5	5	5	4	4	2	2	4	4	2	2	4	4	4	4	4	4	4	4	4	4	4	4	4	4	4	4	4	4	4	4	4	4	4	4	**Ds** **2,3:** **2** **Ds** **4,5:** **4**	**Ds** **2,3:** **4** **Ds** **4,5:** **4**	**Ds** **2,3:** **2** **Ds** **4,5:** **4**	**Ds** **2,3:** **4** **Ds** **4,5:** **4**	2	4	4	4	4	4	4	4	5	5	2	4

Median	**3**	**5**	**3**	**5**	**2**	**4**	**0**	**2**	**3**	**4**	**1**	**2**	**2**	**4**	**3**	**4**	**3**	**4**	**4**	**4**	**2**	**3**	**0**	**2**	**0**	**0**	**1**	**3**	**0**	**0**	**1**	**3**	**1**	**1.5**	**1**	**3**	**1.5**	**3**	**1**	**2.5**	**1**	**1**	**1**	**2**	**0**	**0**	**0**	**0**	**0**	**0**	**0**	**0**
Range	2	1	3	2	2	1	2	4	1	0	3	4	2	1	1	0	1	0	0	0	2	2	4	2	4	4	4	4	4	4	4	4	4	4	4	4	4	4	4	4	3	4	4	4	4	4	4	4	5	5	2	4
Min	3	4	2	3	2	3	0	0	3	4	0	0	2	3	3	4	3	4	4	4	2	2	0	2	0	0	0	0	0	0	0	0	0	0	0	0	0	0	0	0	0	0	0	0	0	0	0	0	0	0	0	0
Max	5	5	5	5	4	4	2	4	4	4	3	4	4	4	4	4	4	4	4	4	4	4	4	4	4	4	4	4	4	4	4	4	4	4	4	4	4	4	4	4	3	4	4	4	4	4	4	4	5	5	2	4

### Proximal versus distal regeneration

Regeneration of the shoulder and elbow muscles was superior to that of the forearm, wrist and finger muscles both before and after surgery. The median muscle powers of the deltoid, rotator cuff, pectoralis major, latissimus dorsi, biceps and triceps ranged from Grades0–4 before surgery and from Grades2–5 after surgery. The median muscle powers of the pronator teres, supinator, the long wrist, finger and thumb extensors and flexors and the intrinsic muscles of the hand ranged from Grades1–2 before surgery and from Grades1–3 after surgery.

### Differential regeneration of muscles supplied by the same nerve

Exemplary for this were the supra- and infraspinatus muscles, both supplied by the suprascapular nerve. Regeneration of the supraspinatus muscle was superior to the infraspinatus, both before and after surgery. Before surgery, the median motor power of the supraspinatus was Grade3 (range:3–4), that of the infraspinatus Grade1 (range:0–3). After surgery, the median motor power of the supraspinatus improved to Grade4 and that of the the infraspinatus to Grade2 (range:0–4). Improvement was recorded in 6 supraspinatus muscles versus 4 infraspinatus muscles

### Differential regeneration of antagonistic muscles

Exemplary for this were the biceps and triceps and the pronator teres and supinator. Before surgery, the median motor power of the biceps was Grade3 (range:3–5), that of the triceps Grade2 (range:2–4). After surgery, the median motor power of the biceps improved to Grade5 (range:4–5), while that of the triceps became Grade3 (range:2–4).

Before surgery, the median motor power of the pronator teres was Grade0 (range:0–4), that of the supinator Grade0 (range:0–4). After surgery, the median motor power of the pronator teres improved to Grade2 (range:2–4), while that of the supinator remained Grade0 (range:0–4).

### Differential regeneration of fibres within the same muscle

**Figure 1:**
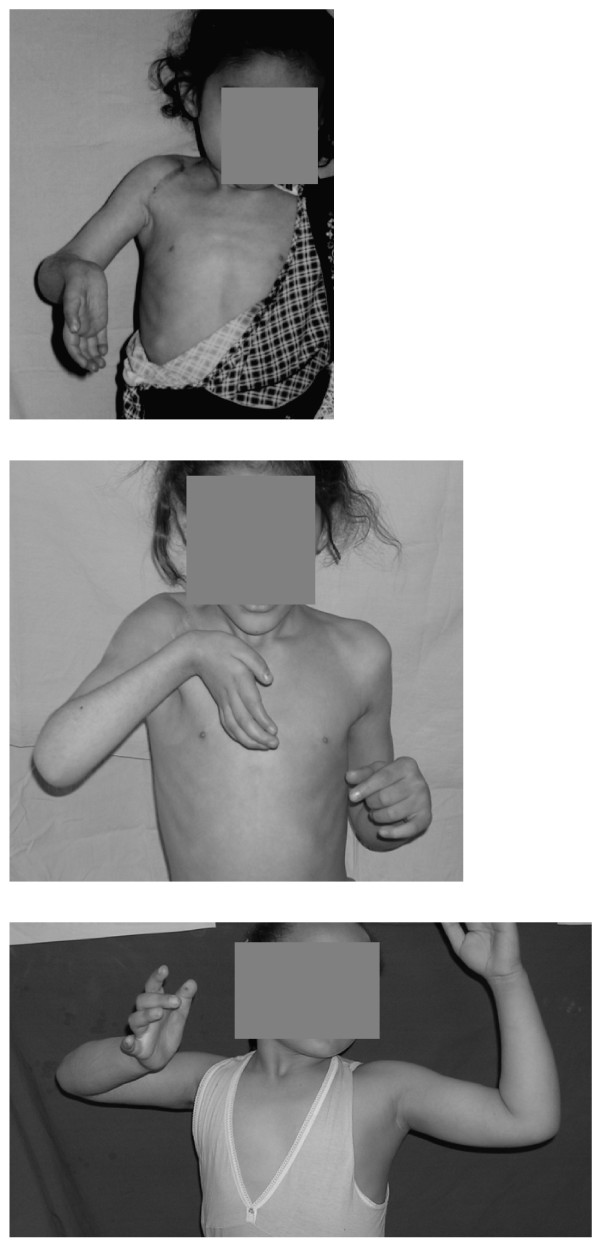
a. Case 1: 1 year after surgery on the right side, no improvement has yet occurred. She was operated upon at the age of 4 for a C5,6 rupture C7,8T1 avulsion, when phrenic to suprascapular and contralateral C7 to lateral, medial and posterior cord neurotization was carried out. The anterior deltoid was Grade3, the lateral deltoid Grade2, the posterior deltoid Grade0. Note the supination deformity of the forearm, the extension deformity at the wrist and biceps cocontraction on attempted active shoulder abduction. At this stage, with that degree of weak shoulder abduction, a humeral external rotation osteotomy or latissimus dorsi to rotatotar cuff transfer will be of no avail. b. Case 1: 2 years after surgery. The anterior deltoid became Grade5, the lateral deltoid Grade4 and the posterior deltoid Grade2. The wrist extensors improved from Grade1 up to Grade3. Some degree of pronation has been regained at the forearm. At this stage, a humeral external rotation osteotomy or latissimus dorsi to rotatotar cuff transfer will also be of no avail, because of extensive biceps cocontraction on attempted shoulder abduction. c. Case7: 4 years after surgery on the right side. She was also operated upon at the age of 4 for a C5,6 rupture C7,8T1 avulsion, when phrenic to suprascapular and contralateral C7 to lateral, medial and posterior cord neurotization was carried out. In addition to improvement of the deltoid and wrist extensors, some shoulder external rotation has been regained as the infraspinatus became Grade3. Biceps cocontraction on attempted shoulder abduction improved. She may therefore benefit from secondary corrective procedures at the shoulder. In addition, a free functional gracilis transplantation has to be carried out to power the weak finger flexors.

Exemplary for this was the deltoid muscle, its anterior and middle fibres regenerating better than its posterior fibres both before and after surgery. Before surgery, the median motor power of the anterior fibres was Grade3 (range:2–5), that of the middle fibres Grade2 (range:2–4) and that of the posterior fibres Grade0 (range:0–2). After surgery, the median motor power of the anterior fibres improved to Grade5 (range:3–5), that of the middle fibres to Grade4 (range:3–4) and that of the posterior fibres to Grade2 (range:0–4) (see Figs 1a and 1b).

### Differential regeneration of muscles having different preoperative motor powers

Exemplary for this were the long wrist, finger and thumb extensors and flexors and the intrinsic muscles of the hand. Out of 53 Grade0 muscles, 47 (88.7%) remained Grade0, 3 (5.7%) improved to Grade1, 3 (5.7%) to Grade2, none to Grades3 or 4. Out of 15 Grade1 muscles, 1 (6.7%) remained Grade1, 6 (40%) improved to Grade2, 8 (53%) to Grade3, none to Grade4. Out of 16 Grade2 muscles, 3 (18.8%) remained Grade2, 4 (25%) improved to Grade3 and 9 (56.3%) improved to Grade4. Out of 10 Grade3 muscles, 7 (70%) remained Grade3 and 3 (30%) improved to Grade4. None of the 11 Grade4 muscles improved to Grade5. Thus Grade1 muscles had a better chance of improving to Grades 1 or 3 and Grade2 muscles to Grades 3 or 4 than Grade0 muscles to Grades 1 or 2.

### Improvement of cocontractions

Cocontractions improved in 3 out of 5 cases (Cases 1, 7 and 8). In Case8, they disappeared completely. In Case1, they disappeared completely on intentional shoulder abduction and flexion and on elbow flexion but remained on unintentionally using the limb. In Case 7, elbow flexion decreased from 130 up to 90 degreees on 90 degree active shoulder abduction (see Fig. 1c); shoulder abduction increased from 60 up to 90 degrees on 90 degree active elbow flexion; cocontractions of the wrist extensors did not improve, however.

### Improvement of deformities

At the shoulder, the internal rotation adduction deformity disappeared in 4 out of 6 patients (Cases1, 6, 7 and 8); Putti's scapular elevation sign became negative. At the forearm, the supination deformity disappeared in all of the 5 cases (Cases1, 3, 5, 6 and 7); the pronation deformity in Case2 persisted, however. At the wrist, due to improvement in extension, the flail wrist assumed a flexible extension deformity in 1 of the 2 cases (Case6); in Case2, the flexor carpi ulnaris, having improved to Grade4, was transferred to the wrist extensors to correct the wrist drop deformity.

### Evaluation scales

The shoulder score improved from 2 to 4 in 3 cases (Cases1, 3 and 6), from 4 to 6 in 2 cases (Cases7 and 8); it remained 2 in 1 case (Case2) and 5 in 2 cases (Cases 4 and 5).

The elbow score improved from 2 to 3 in 1 case (Case2), from 3 to 4 in 2 cases (Cases 1 and 7), from 4 to 5 in 1 case (Case3); it remained 4 in 2 cases (Cases 4 and 6) and 5 in 2 cases (Cases5 and 8).

The hand score improved from 1 to 2 in 3 cases (Cases1, 6 and 7) and from 1 to 3 in 1 case (Case2); it remained 1 in 1 case (Case3), 4 in 1 case (Case4) and 5 in 2 cases (Cases5 and 8).

The pre- and postoperative scores are presented in Table 1.

## Discussion

We have presented our experience in augmenting partially regenerated nerves by end-to-side side-to-side grafting neurotization in late obstetric brachial plexus palsy cases.

Superior proximal to distal regeneration was the first observation. Regeneration of the shoulder and elbow muscles was superior to that of the forearm, wrist and finger muscles. This was consistent with previous reports on early repair of brachial plexus lesions [21,28,30-32]. These reports also advised surgery within 5–6 months after injury. Explanation for this was provided in a morphologic study [35], in which changes within the muscle cells and the motor endplates were the main cause for the poor motor recovery after that time. In our series, however, all but the eighth case were operated upon primarily 3 up to 7 years after injury. The eighth case presented to us 3 years having been operated upon at the age of 3 months. Our aim was to improve already regained muscle power and to activate Grade 0 muscles. For this reason, all nerves to muscles with motor power less than 4 were selected for neurotization. Nerves to muscles with motor power 0 were neurotized, if the electromyogram showed scattered motor unit action potentials on voluntary contraction without interference pattern. This was arbitrarily taken as a sign that the muscle bulk had not been completely replaced by fibrosis and therefore function might be restored to it. This muscle mass preserving effect was recognized by other authors [36]. The median muscle power of the deltoid, rotator cuff, pectoralis major, latissimus dorsi, biceps and triceps improved from Grades0–4 before surgery to Grades2–5 after surgery. This was associated with improved shoulder and elbow scores in 4 out of 8 cases. The median muscle power of the pronator teres, supinator, the long wrist, finger and thumb extensors and flexors and the intrinsic muscles of the hand improved from Grades1–2 before surgery to Grades1–3 after surgery. This was associated with an improved hand score in 4 out of 8 cases. Thus, nerve augmentation might improve already regained muscle power.

Differential regeneration of muscles supplied by the same nerve was the second observation. Exemplary for this were the supra- and infraspinatus muscles, both supplied by the suprascapular nerve. Regeneration of the supraspinatus muscle was superior to the infraspinatus. Superior supraspinatus to infraspinatus regeneration was also observed by other authors after suprascapular nerve grafting or neurotization in the treatment of early brachial plexus lesions [37,38]. In a third study on early repair of obstetric brachial plexus lesions [39], it was concluded that the restoration of a fair range of true glenohumeral external rotation after neurotization of the suprascapular nerve, whether by grafting from C5 or by nerve transfer of the accessory nerve, was disappointingly low.

Differential regeneration of antagonistic muscles was the third observation. Exemplary for this were the biceps and triceps and the pronator teres and supinator. Superior biceps to triceps recovery was observed by other authors [21,40,41]. To account for this, it was noted [42] that fatigue-sensitive afferents inhibited extensor but not flexor motoneurons in humans. In a study on end-to-side neurorrhaphy [43], it was shown that antagonistic nerves had the ability to induce axonal regeneration, but muscle incoordination prevented any useful function. With regard to pronator teres and supinator recovery, in a historical cohort of obstetric brachial plexus lesions, it was observed that external rotation and supination were the last to recover and recovered the least [44].

Differential regeneration of fibres within the same muscle was the fourth observation. Exemplary for this was the deltoid muscle, its anterior and middle fibres regenerating better than its posterior fibres both before and after surgery. In a retrospective study of 33 traumatic lesions of the axillary nerve [45], deltoid muscle strength was noted to be good or fair in 18 patients and poor in 15. The outcome seemed to be better in isolated lesions than in complex nerve lesions, in patients younger than 25 years compared to older patients, in patients treated with neurolysis compared to grafting, and when graft length was. The outcome was less favourable when associated osteoarticular lesions were present and when surgery was delayed beyond six months. In another study [46], good or very good deltoid function was obtained in 23 out of 25 direct repairs of isolated axillary lesions, and in all 4 patients with associated injury to the musculocutaneous nerve. Only 4 good results were obtained in the 8 patients who also had injuries to the suprascapular nerve. In both of these studies no mention was made as to the regeneration of the individual parts of the deltoid muscle. In an anatomic study of the internal topographic features of the axillary nerve [47], however, the axillary nerve was divided into three segments. Proximal to the subscapularis muscle, the axillary nerve formed a single nerve trunk. Nerve fascicles to the deltoid muscle were identified at its lateral part. In front of the subscapularis muscle, the axillary nerve formed into the lateral and medial fasciculi groups. Distal to the subscapularis muscle, the nerve divided into anterior and posterior branches, which were continuations of the lateral and medial fasciculi groups, respectively. The anterior branch contained all fibers that innervated the anterior and middle deltoid muscle. In 90% of cases, the posterior branch containsed part or all nerve fibers to the posterior deltoid muscle. Nerve fibers to the teres minor and cutaneous sensory fibers were found in the posterior branch. It was concluded, that in neurotization of the deltoid muscle, the best approach was to match the donor nerve to the lateral fasciculi group, which would give the highest percentage of reinnervation of the deltoid muscle. In a fourth study [48], it was concluded that secondary compression of the axillary nerve in the quadrangular space was a separate and common reason for impairment in children with brachial plexus birth palsy and persistent weakness of the deltoid muscle and might provide an important reason for early intervention.

Differential regeneration of muscles having different preoperative motor powers was the fifth observation. Exemplary for this were the long wrist, finger and thumb extensors and flexors and the intrinsic muscles of the hand. Grade1 muscles had a better chance of improving to Grades 1 or 3 and Grade2 muscles to Grades 3 or 4 than Grade0 muscles to Grades 1 or 2. Thus functional improvement was primarily expected in Grade2 muscles. This is supported by the experimental observation [35] that, in long lasting pre-suture denervation intervals, changes within the muscle cells and the motor endplates take place and are of outstanding importance for the poor motor recovery. Especially after late nerve sutures the arrival of axons within the muscle is by no means necessarily followed by a sufficient recurrence of its function. An interesting speculation is the role of the muscle target organ as a promoting factor for nerve fibre regeneration in nerve grafts, whether higher grade muscles are expected to promote axonal growth more than lower grade muscles. This was studied in rabbits, sheep and humans [49]. Excellent regeneration of myelinated nerve fibres was observed without target organ influence through the whole length of the nerve graft, with an increase in the number of nerve fibres up to fourfold at the distal end. In the sheep series the additional contact with a muscle target organ for 6 months had a variable effect on the fibre population in the distal end of the nerve graft. In humans, however, a decrease of regenerating nerve fibres arriving at the distal end of nerve grafts was noted. Interestingly, a possible role of the muscle target organ as a promoting factor for nerve fibre regeneration in nerve grafts came from biomaterial research, where muscle-derived protein with molecular mass of 77 kDa (MDP77) in artificial nerve grafts was shown to promote motor nerve regeneration [50,51].

Improvement of cocontractions was the sixth observation. Cocontractions improved in 3 out of 5 cases. In a clinical study [52], cocontractions were classified into the following types: TypeI involving the deltoid and biceps muscles, TypeII involving the deltoid, biceps and triceps muscles, TypeIII involving the biceps and triceps muscles, TypeIV involving the deltoid, biceps, triceps and forearm muscles, TypeV involving the deltoid, biceps and forearm muscles, TypeVI involving the biceps, triceps and forearm muscles and TypeVII involving the triceps and forearm muscles. Cocontractions did not improve, but physical therapy or operative treatment brought improvement in daily activities. Clinical severity of cross-reinnervation was correlated to the severity of paralysis and in proportion to the ratio of normally recovered nerve fibers and cross-reinnervated nerve fibers. In our study, cocontractions were TypeI in 4 cases, TypeV in 1 case. Both this study and the improvement of cocontractions in our study lend support to the hypothesis that cocontractions are due to lack of collateral rather than axial axonal sprouting.

Improvement of deformities was the seventh observation. At the shoulder, in 4 out of 6 patients the internal rotation adduction deformity disappeared; Putti's scapular elevation sign became negative. This observation is consistent with other reports [53]. At the forearm, the supination deformity disappeared; the pronation deformity persisted, however. At the wrist, due to improvement in extension, the flail wrist assumed a flexible extension deformity in 1 of the 2 cases; in a further case, the flexor carpi ulnaris, having improved to Grade4, was transferred to the wrist extensors to correct the wrist drop deformity.

In conclusion, nerve augmentation of late brachial plexus injuries is expected to improve muscle power in the biceps, pectoral muscles, supraspinatus, anterior and lateral deltoids, triceps and in forearm muscles with motor power Grade2 or more. It is also expected to improve cocontractions. It is less expected to improve infraspinatus power. Therefore, after recovery of deltoid function, patients should undergo a humeral derotation osteotomy and a tendon transfer (see Figs 1a,1b and 1c). As it is less expected to improve Grade0 or 1 forearm muscles, these should be powered with a free muscle transfer [54]. But the surgeon needn't use nerve grafts. The median, ulnar and radial nerves may act as bridges for neurotization. This was tried out experimentally [55] and confirmed clinically [56]. For the same reason and contrary to other reports [54,57], the transplanted muscle can be placed at the forearm. Inspite of all of the above, the results obtained are still inferior to those expected clinically. First, we need to revise our end-to-side techniques. The channel carrying capacity of the donor nerve, donor-recipient neurorrhaphy and the augmented recipient has to be increased by cotrophism [58], cotropism [59-62] and cotransplantation [63-68]. Second, restoration of recipient muscle mass or regenerative potential should be aimed at [69-71].

Finally, this study has several limitations. First, the sample size is small, consisting only of 8 cases. Second, there are no controls. These are necessary to rule out any natural improvement of the lesion. Third, although we have tried to increase muscle testing reliability through documenting it on both limbs by digital photographs, there is still marked intra- and interobserver variability in testing muscle power and cocontractions.

## References

[B1] Kay SPJ Obstetrical brachial palsy. Review article Br J Plast Surg 1998 1 43 50 9577318 10.1054/bjps.1997.0166

[B2] Noetzel MJ Park TS Robinson S Kaufman B Prospective study of recovery following neonatal brachial plexus injury J Child Neurol 2001 1 7 488 492 11453444 10.1177/088307380101600705

[B3] Hoeksma AF ter Steeg AM Nelissen RG van Ouwerkerk WJ Lankhorst GJ de Jong BA Neurological recovery in obstetric brachial plexus injuries: an historical cohort study Dev Med Child Neurol 2004 1 2 76 83 10.1017/S0012162204000179 14974631

[B4] O'Brien DF Park TS Noetzel MJ Weatherly T Management of birth brachial plexus palsy Childs Nerv Syst 2006 1 2 103 112 10.1007/s00381-005-1261-y 16320018

[B5] Haerle M Gilbert A Management of complete obstetric brachial plexus lesions J Pediatr Orthop 2004 1 2 194 200 15076607 10.1097/00004694-200403000-00012

[B6] Gilbert A Pivato G Kheiralla T Long-term results of primary repair of brachial plexus lesions in children Microsurgery 2006 1 4 334 342 10.1002/micr.20248 16634084

[B7] Birch R Ahad N Kono H Smith S Repair of obstetric brachial plexus palsy: results in 100 children J Bone Joint Surg Br 2005 1 8 1089 1095 10.1302/0301-620X.87B8.15975 16049245

[B8] Grossman JA Price AE Tidwell MA Ramos LE Alfonso I Yaylali I Outcome after later combined brachial plexus and shoulder surgery after birth trauma J Bone Joint Surg Br 2003 1 8 1166 1168 10.1302/0301-620X.85B8.14246 14653601

[B9] Partridge C Edwards S Obstetric brachial plexus palsy: increasing disability and exacerbation of symptoms with age Physiother Res Int 2004 1 4 157 163 10.1002/pri.319 15790253

[B10] Waters PM Bae DS The effect of derotational humeral osteotomy on global shoulder function in brachial plexus birth palsy J Bone Joint Surg Am 2006 1 5 1035 1042 10.2106/JBJS.E.00680 16651578

[B11] Aydin A Ozkan T Onel D Does preoperative abduction value affect functional outcome of combined muscle transfer and release procedures in obstetrical palsy patients with shoulder involvement? BMC Musculoskelet Disord 1 25 2004 Aug 3 10.1186/1471-2474-5-25 15291961 PMC514557

[B12] Kirkos JM Kyrkos MJ Kapetanos GA Haritidis JH Brachial plexus palsy secondary to birth injuries J Bone Joint Surg Br 2005 1 2 231 235 10.1302/0301-620X.87B2.14739 15736749

[B13] Pagnotta A Haerle M Gilbert A Long-term results on abduction and external rotation of the shoulder after latissimus dorsi transfer for sequelae of obstetric palsy Clin Orthop Relat Res 2004 426 199 205 10.1097/01.blo.0000138957.11939.70 15346074

[B14] Kambhampati SB Birch R Cobiella C Chen L Posterior subluxation and dislocation of the shoulder in obstetric brachial plexus palsy J Bone Joint Surg Br 2006 1 2 213 219 10.1302/0301-620X.88B2.17185 16434527

[B15] Viterbo F Teixeira E Hoshino K Padovani CR End-to-side neurorrhaphy with and without perineurium Sao Paulo Med J 1998 1 5 1808 1814 10.1590/S1516-31801998000500005 10030106

[B16] Isaacs J Allen D Chen LE Nunley J 2nd Reverse end-to-side neurotization J Reconstr Microsurg 2005 1 1 43 8 discussion 49–50 10.1055/s-2005-862780 15672319

[B17] Kerns JM Sladek EH Malushte TS Bach H El-Hassan B Kitidumrongsook P Kroin JS Shott S Gantsoudes G Gonzalez MH End-to-side nerve grafting of the tibial nerve to bridge a neuroma-in-continuity Microsurgery 2005 1 2 155 164 discussion 164–166 10.1002/micr.20096 15712214

[B18] Grossman JA DiTaranto P Yaylali I Alfonso I Ramos LE Price AE Shoulder function following late neurolysis and bypass grafting for upper brachial plexus birth injuries J Hand Surg [Br] 2004 1 4 356 358 10.1016/j.jhsb.2004.03.008 15234499

[B19] Amr SM Moharram AN Repair of brachial plexus lesions by end-to-side side-to-side grafting neurorrhaphy: experience based on 11 cases Microsurgery 2005 1 2 126 146 10.1002/micr.20036 15389968

[B20] Chow BCL Blaser S Clarke HM Predictive value of computed tomographic myelography in obstetrical brachial plexus palsy Plast Reconstr Surg 2000 1 971 977 10.1097/00006534-200010000-00001 11039366

[B21] Terzis JK Papakonstantinou KC The surgical treatment of brachial plexus injuries in adults Plast Reconstr Surg 2000 1 1097 1122 10.1097/00006534-200010000-00022 11039383

[B22] Hierner R Becker M Berger A Indications and results of operative treatment in birth-related brachial plexus injuries Handchir Mikrochir Plast Chir 2005 1 5 323 331 10.1055/s-2005-865898 16287017

[B23] Millesi H Meissl G Berger A Further experience with interfascicular grafting of the median, ulnar and radial nerves J Bone Joint Surg (Am) 1981 1 209 218 767344

[B24] Arcand MA Reider B Reider B Shoulder and upper arm The orthopaedic physical examination 2004 2 Philadelphia, Elsevier 17 66

[B25] Tennent TD Beach WR Meyers JF A review of the special tests associated with shoulder examination. Part I: the rotator cuff tests Am J Sports Med 2003 1 1 154 160 12531773 10.1177/03635465030310011101

[B26] Richards DP Burkhart SS Lo IK Subscapularis tears: arthroscopic repair techniques Orthop Clin North Am 2003 1 4 485 498 Review 10.1016/S0030-5898(03)00096-8 14984188

[B27] McFarland EG Selhi HS Keyurapan E Clinical evaluation of impingement: what to do and what works J Bone Joint Surg Am 2006 1 432 441 16475277 10.2106/00004623-200602000-00026

[B28] Amr SM Traumatic brachial plexus palsy; a report of 30 cases Medical Journal of Cairo University 2000 1 715 730

[B29] Hattori Y Doi K Toh S Baliarsing AS Surgical approach to the spinal accessory nerve for brachial plexus reconstruction J Hand Surg [Am] 2001 1 6 1073 1076 10.1053/jhsu.2001.28762 11721253

[B30] Hentz VR Green DP, Hotchkiss RN Microneural reconstruction of the brachial plexus Operative hand surgery 1993 New York Edinburgh London Melbourne Tokyo: Churchill-Livingstone 1223 1252

[B31] Leffert RD Green DP, Hotchkiss RN Brachial plexus Operative hand surgery 1993 New York Edinburgh London Melbourne Tokyo: Churchill-Livingstone 1483 1516

[B32] Millesi H Chirurgie der peripheren Nerven Spezieller Teil, 2. Plexus brachialis und seine Aeste 1992 Muenchen Wien Baltimore: Urban&Schwarzenberg 79 112

[B33] Mcguiness CN Kay SP The prespinal route in contralateral C7 nerve root transfer for brachial plexus avulsion injuries J Hand Surg [Br] 2002 1 2 159 160 10.1054/jhsb.2001.0665 12027492

[B34] Oberlin C Ameur NE Teboul F Beaulieu JY Vacher C Restoration of elbow flexion in brachial plexus injury by transfer of ulnar nerve fascicles to the nerve to the biceps muscle Tech Hand Up Extrem Surg 2002 1 2 86 90 10.1097/00130911-200206000-00007 16520622

[B35] Richter HP Impairment of the restoration of motor function after delayed nerve suture Fortschr Med 1 10 414 418 1982 Mar 11 7040185

[B36] Oswald TM Zhang F Lei MP Gerzenshtein J Lineaweaver WC Muscle flap mass preservation with end-to-side neurorrhaphy: an experimental study J Reconstr Microsurg 2004 1 6 483 488 10.1055/s-2004-833504 15356771

[B37] Malessy MJ de Ruiter GC de Boer KS Thomeer RT Evaluation of suprascapular nerve neurotization after nerve graft or transfer in the treatment of brachial plexus traction lesions J Neurosurg 2004 1 3 377 389 15352593 10.3171/jns.2004.101.3.0377

[B38] Leechavengvongs S Witoonchart K Uerpairojkit C Thuvasethakul P Malungpaishrope K Combined nerve transfers for C5 and C6 brachial plexus avulsion injury J Hand Surg [Am] 2006 1 2 183 189 10.1016/j.jhsa.2005.09.019 16473676

[B39] Pondaag W de Boer R van Wijlen-Hempel MS Hofstede-Buitenhuis SM Malessy MJ External rotation as a result of suprascapular nerve neurotization in obstetric brachial plexus lesions Neurosurgery 2005 1 3 530 537 10.1227/01.NEU.0000170557.13788.D2 16145533

[B40] Martin PG Smith JL Butler JE Gandevia SC Taylor JL Fatigue-sensitive afferents inhibit extensor but not flexor motoneurons in humans J Neurosci 1 18 4796 4802 2006, May 3 10.1523/JNEUROSCI.5487-05.2006 16672652 PMC6674170

[B41] Kandenwein JA Kretschmer T Engelhardt M Richter HP Antoniadis G Surgical interventions for traumatic lesions of the brachial plexus: a retrospective study of 134 cases J Neurosurg 2005 1 4 614 621 16266042 10.3171/jns.2005.103.4.0614

[B42] Bentolila V Nizard R Bizot P Sedel L Complete traumatic brachial plexus palsy. Treatment and outcome after repair J Bone Joint Surg Am 1999 1 1 20 28 9973050 10.2106/00004623-199901000-00004

[B43] Lutz BS Chuang DC Hsu JC Ma SF Wei FC Selection of donor nerves – an important factor in end-to-side neurorrhaphy Br J Plast Surg 2000 1 2 149 154 10.1054/bjps.1999.3252 10878839

[B44] Hoeksma AF ter Steeg AM Nelissen RG van Ouwerkerk WJ Lankhorst GJ de Jong BA Neurological recovery in obstetric brachial plexus injuries: an historical cohort study Dev Med Child Neurol 2004 1 2 76 83 10.1017/S0012162204000179 14974631

[B45] Wehbe J Maalouf G Habanbo J Chidiac RM Braun E Merle M Surgical treatment of traumatic lesions of the axillary nerve. A retrospective study of 33 cases Acta Orthop Belg 2004 1 1 11 18 15055312

[B46] Zhao X Hung LK Zhang GM Lao J Applied anatomy of the axillary nerve for selective neurotization of the deltoid muscle Clin Orthop Relat Res 2001 390 244 251 10.1097/00003086-200109000-00028 11550872

[B47] Alnot JY Valenti P Surgical repair of the axillary nerve. Apropos of 37 cases Int Orthop 1991 1 1 7 11 10.1007/BF00210524 2071286

[B48] Adelson PD Nystrom NA Sclabassi R Entrapment neuropathy contributing to dysfunction after birth brachial plexus injuries J Pediatr Orthop 2005 1 5 592 597 10.1097/01.bpo.0000161092.23468.1c 16199937

[B49] Frey M Koller R Liegl C Happak W Gruber H Role of a muscle target organ on the regeneration of motor nerve fibres in long nerve grafts: a synopsis of experimental and clinical data Microsurgery 1996 1 2 80 88 10.1002/(SICI)1098-2752(1996)17:2<80::AID-MICR2>3.0.CO;2-# 8914062

[B50] Itoh S Fujimori KE Uyeda A Matsuda A Kobayashi H Shinomiya K Tanaka J Taguchi T Long-term effects of muscle-derived protein with molecular mass of 77 kDa (MDP77) on nerve regeneration J Neurosci Res 1 5 730 738 2005 Sep 1 10.1002/jnr.20582 16007679

[B51] Itoh S Uyeda A Hukuoka Y Fujimori KE Matsuda A Ichinose S Kobayashi H Shinomiya K Tanaka J Taguchi T Muscle-specific protein MDP77 specifically promotes motor nerve regeneration in rats Neurosci Lett 1 3 175 177 2004 Apr 29 10.1016/j.neulet.2004.02.058 15082161

[B52] Yagi I Clinical study of cross-reinnervation in obstetrical paralysis Nippon Seikeigeka Gakkai Zasshi 1984 1 8 761 778 6501985

[B53] Birch R Ahad N Kono H Smith S Repair of obstetric brachial plexus palsy: results in 100 children J Bone Joint Surg Br 2005 1 8 1089 1095 10.1302/0301-620X.87B8.15975 16049245

[B54] Hattori Y Doi K Ikeda K Pagsaligan JM Watanabe M Restoration of prehension using double free muscle technique after complete avulsion of brachial plexus in children: a report of three cases J Hand Surg [Am] 2005 1 4 812 819 10.1016/j.jhsa.2004.12.011 16039378

[B55] Shah MH Kasabian AK Karp NS Kolker AR Dublin BA Zhang L Sakuma J Axonal regeneration through an autogenous nerve bypass: an experimental study in the rat Ann Plast Surg 1997 1 4 408 414 discussion 414-5 10.1097/00000637-199704000-00017 9111903

[B56] McCallister WV Cober SR Norman A Trumble TE Using intact nerve to bridge peripheral nerve defects: an alternative to the useof nerve grafts J Hand Surg [Am] 2001 1 2 315 325 10.1053/jhsu.2001.22918 11279579

[B57] Doi K New reconstructive procedure for brachial plexus injury Clinics in Plastic Surgery 1997 1 1 75 85 9211030

[B58] McConnell MP Dhar S Naran S Nguyen T Bradshaw RA Evans GR In vivo induction and delivery of nerve growth factor, using HEK-293 cells Tissue Eng 2004 1 9–10 1492 1501 15588408 10.1089/ten.2004.10.1492

[B59] Itoh S Matsuda A Kobayashi H Ichinose S Shinomiya K Tanaka J Effects of a laminin peptide (YIGSR) immobilized on crab-tendon chitosan tubes on nerve regeneration J Biomed Mater Res B Appl Biomater in press 2005 Mar 7 15754343 10.1002/jbm.b.30224

[B60] Belkas JS Shoichet MS Midha R Peripheral nerve regeneration through guidance tubes Neurol Res 2004 1 2 151 160 10.1179/016164104225013798 15072634

[B61] Fansa H Keilhoff G Comparison of different biogenic matrices seeded with cultured Schwann cells for bridging peripheral nerve defects Neurol Res 2004 1 2 167 173 10.1179/016164104225013842 15072636

[B62] Nishiura Y Brandt J Nilsson A Kanje M Dahlin LB Addition of cultured Schwann cells to tendon autografts and freeze-thawed muscle grafts improves peripheral nerve regeneration Tissue Eng 2004 1 1–2 157 164 10.1089/107632704322791808 15009941

[B63] Komiyama T Nakao Y Toyama Y Vacanti CA Vacanti MP Ignotz RA Novel technique for peripheral nerve reconstruction in the absence of an artificial conduit J Neurosci Methods 1 2 133 140 2004 Apr 30 10.1016/j.jneumeth.2003.11.020 15003379

[B64] Reier PJ Cellular Transplantation Strategies for Spinal Cord Injury and Translational Neurobiology Neurorx 2004 1 4 424 451 10.1602/neurorx.1.4.424 15717046 PMC534951

[B65] Mimura T Dezawa M Kanno H Sawada H Yamamoto I Peripheral nerve regeneration by transplantation of bone marrow stromal cell-derived Schwann cells in adult rats J Neurosurg 2004 1 5 806 812 15540919 10.3171/jns.2004.101.5.0806

[B66] Heine W Conant K Griffin JW Hoke A Transplanted neural stem cells promote axonal regeneration through chronically denervated peripheral nerves Exp Neurol 2004 1 2 231 240 10.1016/j.expneurol.2004.06.014 15380475

[B67] Tohill M Terenghi G Stem-cell plasticity and therapy for injuries of the peripheral nervous system Biotechnol Appl Biochem 2004 1 Pt 1 17 24 15270703 10.1042/BA20030173

[B68] Dezawa M Kanno H Hoshino M Cho H Matsumoto N Itokazu Y Tajima N Yamada H Sawada H Ishikawa H Mimura T Kitada M Suzuki Y Ide C Specific induction of neuronal cells from bone marrow stromal cells and application for autologous transplantation J Clin Invest 2004 1 12 1701 1710 10.1172/JCI200420935 15199405 PMC420509

[B69] Sarig R Baruchi Z Fuchs O Nudel U Yaffe D Regeneration and transdifferentiation potential of muscle-derived stem cells propagated as myospheres. When inoculated into injured muscle, myosphere-derived cells participated in regeneration, forming multinucleated cross-striated mature fibers Stem Cells 2006 1 7 1769 1778 10.1634/stemcells.2005-0547 16574751

[B70] Vilquin JT Myoblast transplantation: clinical trials and perspectives Mini-review Acta Myol 2005 1 2 119 127 16550929

[B71] Matziolis G Winkler T Schaser K Wiemann M Krocker D Tuischer J Perka C Duda GN Autologous bone marrow-derived cells enhance muscle strength following skeletal muscle crush injury in rats Tissue Eng 2006 1 2 361 367 10.1089/ten.2006.12.361 16548694

